# Reporting of colorectal anastomotic leakage: Worldwide audit of national databases

**DOI:** 10.1111/codi.70619

**Published:** 2026-09-07

**Authors:** Anne de Wit, Freek Daams, Katherine Lam, Katherine Donovan, Daniela Ramos‐Delgado, Danique J. I. Heuvelings, Nicole D. Bouvy, Nader Francis, Marylise Boutros, Patricia Sylla

**Affiliations:** ^1^ Department of Surgery Amsterdam University Medical Center Amsterdam The Netherlands; ^2^ Cancer Center Amsterdam Amsterdam The Netherlands; ^3^ Department of Surgery Mount Sinai Hospital New York New York USA; ^4^ Icahn School of Medicine at Mount Sinai New York New York USA; ^5^ Department of Surgery Maastricht University Medical Center Maastricht The Netherlands; ^6^ Department of Surgery Leiden University Medical Center Leiden The Netherlands; ^7^ Department of Surgery and Interventional Science University College London UK; ^8^ The Griffin Institute Northwick Park and St Mark's Hospital Harrow UK; ^9^ Department of Surgery Cleveland Clinic Florida Weston Florida USA

**Keywords:** anastomotic leakage, colorectal surgery, database, reporting

## Abstract

**Aim:**

Reporting of colorectal anastomotic leakage (CAL) risk factors and outcomes is not standardized worldwide. This study searched a cohort of national surgical registries to assess the scope of CAL‐related elements that are routinely reported.

**Methods:**

This international audit was conducted between June 2025 and April 2026. Surgeons were contacted to determine whether their country maintained a national registry and to provide the list of CAL‐related variables collected. Registry variables were compared to the 79 CoReAL (Consensus for Reporting of colorectal Anastomotic Leaks) variables, an evidence‐based framework developed to standardize how key CAL‐related variables are reported. The primary outcome was the proportion of countries with a national registry reporting CAL and CAL‐associated variables. The secondary outcome was the proportion and type of CoReAL variables included across registries.

**Results:**

Thirty‐eight countries across all continents participated. Among these, seven countries (18.4%) maintain national registries that centrally capture post‐operative outcomes. Across all registries, the median number of registered variables was 28 (range 18–37, IQR 4). Completeness differed significantly between countries, with 10 elements being captured by all (*p* = 0.003). Registries reported a median of 11/25 preoperative (8–19, 6), 6/16 intraoperative (3–8, 2), 8/16 early post‐operative (5–12, 4) and 2/22 (0–13, 4) of the long‐term post‐operative elements.

**Conclusion:**

The majority of countries lack a national registry for systematic CAL reporting. Among existing registries, substantial heterogeneity in reported variables was observed, including CAL risk factors and outcomes, particularly beyond 30 days. We propose global adoption of the CoReAL reporting framework to fill this gap in quality reporting of CAL.

**What does this paper add to the literature?**

Most countries lack national registries for colorectal anastomotic leakage, and existing registries incompletely capture key risk factors and outcomes. This first global assessment identifies major deficiencies and heterogeneity in current reporting practices, highlighting the need for standardized data collection to improve patient care, quality assurance and international benchmarking.

## INTRODUCTION

Assessment of risk factors for colorectal anastomotic leakage (CAL) and the documentation of CAL‐related outcomes remain inconsistently performed across healthcare systems worldwide [1, 2, 3]. Despite advances in colorectal surgery, there is still considerable variation in how institutions identify, record, and monitor factors associated with CAL. This lack of standardization limits the ability to accurately evaluate patient risk profiles and undermines efforts to implement evidence‐based preventive strategies [4, 5].

The absence of uniform reporting practices has important clinical implications. Without reliable and comprehensive data, surgeons may face challenges in preoperative planning and intraoperative decision‐making [6, 7]. Furthermore, incomplete documentation of CAL‐related outcomes can contribute to delays in diagnosis and treatment, potentially increasing patient morbidity, prolonging hospital stays and placing additional strain on healthcare resources [8, 9].

National surgical registries have the potential to play a pivotal role in improving the consistency and quality of CAL reporting [10, 11]. By systematically collecting data on patient characteristics, surgical techniques and post‐operative outcomes, these registries can provide valuable insights into patterns of care and areas for improvement. However, the extent to which CAL‐related variables are currently captured across different registries remains unclear.

Therefore, the present study searched a cohort of national surgical registries to evaluate the scope and consistency of CAL‐related elements that are routinely reported. By the identification of existing variations and potential deficiencies in data collection, this work aimed to guide future efforts towards standardized reporting and ultimately enhance the quality of care for patients undergoing colorectal surgery.

## METHOD

### Study design

This study was an audit of national surgical registries across all continents. The data collection was conducted between June 2025 and April 2026. The study protocol was approved by the Mount Sinai Hospital Institutional Review Board. As no individual patient data were collected or analysed, the study was declared exempt from the requirements of the Medical Research Involving Human Subjects Act (WMO).

### Participants

All available national surgical registries worldwide were considered for inclusion in this study. During the inclusion period, surgeons were identified through CAL‐related publications on Pubmed, conference participation and enrolment in surgical associations, and subsequently invited to participate. They were asked whether their country maintained a national registry for CAL (and other post‐operative outcomes) and, if so, to provide the detailed list of the CAL‐related variables collected. In the absence of a national registry, respondents were asked whether institutional‐ or regional‐level systems existed for capturing such data.

At no point during the study were individual patient data collected or analysed. Instead, the assessment was limited to the structural characteristics of the registries, including the names of variables, their definitions, units of measurement and available response options. All collected information was translated from the local language into English using an online translation tool.

To be eligible for inclusion, a registry had to be established at the national level and collect data from hospitals across the respective country. In addition, it had to include data on post‐operative outcomes and explicitly capture the occurrence of CAL. Registries with varying scopes were included, regardless of whether they focused exclusively on oncological patients or encompassed all colorectal surgical cases. Similarly, registries were eligible whether they recorded colon and rectal resections separately or in a combined manner. Registries could be administered either by individual hospitals or by independent organizations and could be established by governmental bodies, insurance providers, cancer programmes or quality improvement initiatives. Registries were excluded if they were not implemented on a national scale or if they lacked data on post‐operative outcomes including CAL.

### Outcomes

The primary outcome of this study was the proportion of countries that maintained a national registry reporting CAL and CAL‐associated variables. These proportions were additionally stratified by continent to assess geographic variation. The definition and grading of CAL were not standardized upfront but were instead collected as reported within each registry during the data collection.

The secondary outcome was the proportion and types of CAL‐associated variables included across the identified registries. Reported variables were systematically compared with the 79 variables defined in the CoReAL (Consensus for Reporting of colorectal Anastomotic Leaks) framework, an evidence‐based initiative developed to standardize the reporting of CAL‐related variables [12]. These variables were categorized into preoperative (demographics, disease details, surgery preparation details), intraoperative (surgery details, intraoperative pitfalls, intraoperative difficulty), 30 or 90 days post‐operative (discharge, anastomotic complications) and long‐term post‐operative (anastomotic complications) periods. Proportions of reported variables were also analysed within each of these periods.

### Statistical analysis

Collected study data were analysed using Excel (Microsoft, Version 2501, Redmond (WA, USA)) and Statistical Package for the Social Sciences (IBM SPSS Statistics, Version 28.0, Armonk (NY, USA)). Descriptive statistical analyses were performed to examine study outcomes, including a Fisher's exact test to compare completeness of all variable categories. Results were presented as proportions for categorical variables and as means with standard deviations (SD) or medians with interquartile ranges (IQR) for continuous variables, depending on data distribution. A p‐value of <0.05 was considered statistically significant.

## RESULTS

### National registries

**FIGURE 1:**
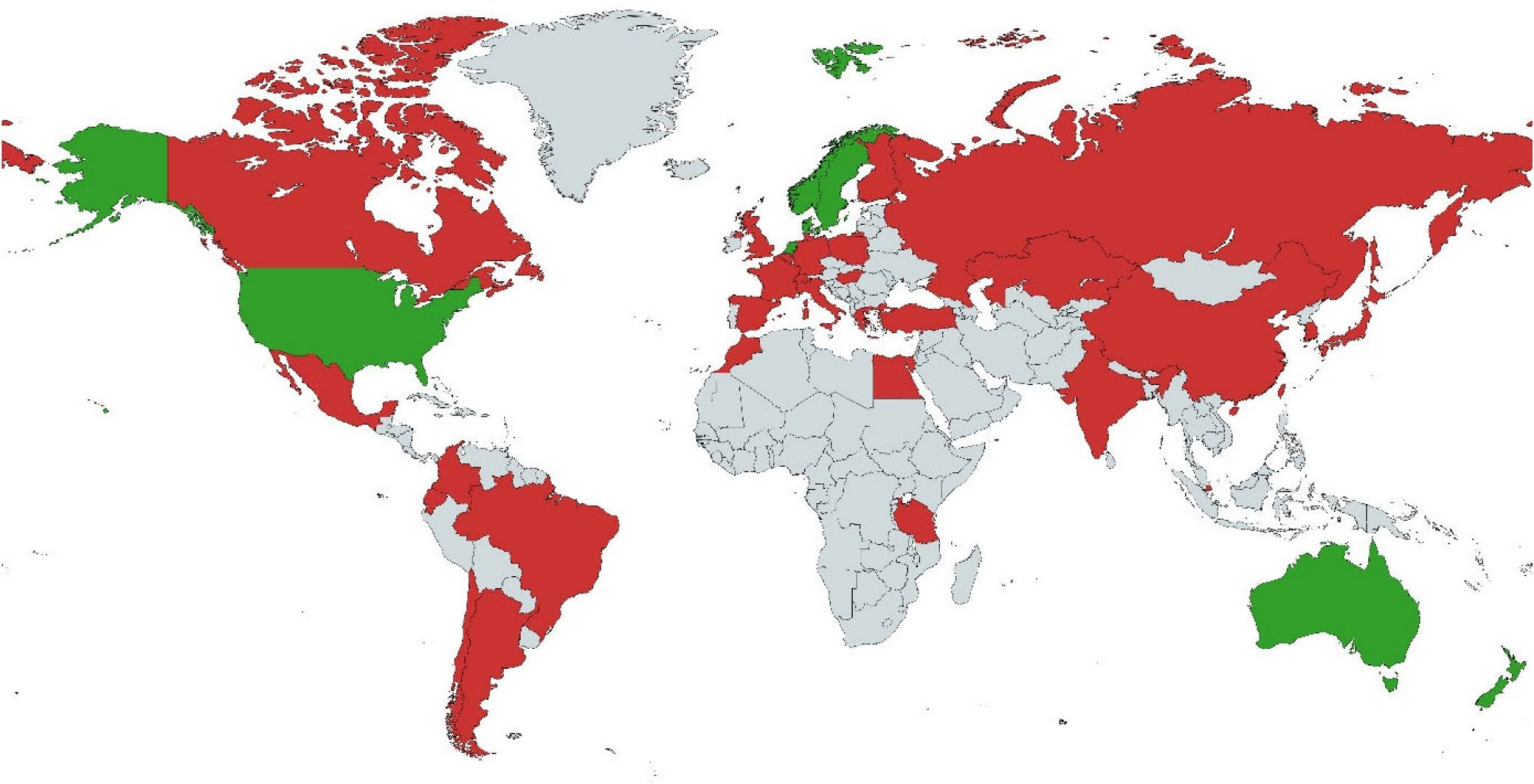
Countries with national registries reporting post‐operative complications, including CAL. Green (national registry): Australia, Denmark, the Netherlands, New Zealand, Norway, Sweden, United States of America. Red (no national registry): Argentina, Belgium, Brazil, Canada, Chile, China, Colombia, Ecuador, Egypt, Finland, France, Germany, Greece, Hong Kong, Hungary, India, Italy, Japan, Kazakhstan, Mexico, Morocco, Poland, Russia, Singapore, South Korea, Spain, Switzerland, Taiwan, Tanzania, Turkey, United Kingdom. Grey: Countries with no available information.

A total of 38 countries were included in this study, representing all continents (3 in North America, 5 in South America, 15 in Europe, 3 in Africa, 10 in Asia and 2 in Oceania). Among these, seven countries (18.4%) maintained national registries that centrally capture post‐operative outcomes and complications after colorectal surgery: Australia, Denmark, the Netherlands, New Zealand, Norway, Sweden and the United States of America (Figure 1) [13, 14, 15, 16, 17, 18]. When stratified by continent, 33.3% of countries in North America surveyed a registry, compared to 26.6% in Europe and 100% in Oceania, while no national registries reporting CAL were identified in South America, Africa and Asia. Two identified surgeons from each of 19 additional countries across all continents were contacted but did not respond to our inquiries.

The identified national registries were either inclusive of all types of CAL (6/7) or specific to either colon or rectal anastomotic complications (1/7). In all cases, data were entered electronically at the level of individual hospitals and subsequently aggregated and stored centrally under the supervision of national surgical associations and governmental bodies. National registries were primarily used for quality control, but data are also accessed for research (Table 1).

**TABLE 1:** National registries.

Country	Name of registry	Version	Separate colon and rectum registry	Length post‐operative follow‐up	Length of long‐term follow‐up
Australia	BCOR	01‐01‐2017	Merged	30 days	Unknown
Denmark	DCCG	25‐10‐2022	Merged	90 days	Unknown
Netherlands	DCRA	01‐01‐2025	Merged	90 days	Unknown
New Zealand	BCOR	01‐01‐2017	Merged	30 days	Unknown
Norway	NORGAST	31–12‐2023	Merged (incl. HPB and UGI)	30 days (90 days mortality)	–
Sweden	INCA	12‐2‐2024	Merged	30 days	5 years
United States	NSQIP	1‐1‐2025	Separate	30 days	–

In countries without national registries, alternative data collection systems were identified, though these varied substantially in scope and structure. Some countries reported the existence of national registries; however, these did not include variables related to CAL. Other countries confirmed the absence of a national registry altogether but indicated that institutional databases were maintained, often with broad and heterogeneous data collection practices.

### Element capturing

Across all included registries, the median number of documented variables was 28 (range 18–37, IQR 4). The overall completeness of registries was 36.2%. Sweden demonstrated the highest completeness, capturing 46.8% of the variables within its registry. In comparison, overall completeness was 35.4% in Australia, 35.4% in Denmark, 40.5% in the Netherlands, 22.8% in Norway, 35.4% in New Zealand and 36.7% in the United States of America. Completeness differed significantly between countries, with 10 variables consistently captured across all registries (*p* = 0.003) (Table 2). The only universally recorded variable not included in the CoReAL framework was patient age.

**TABLE 2:** Element capturing across countries.

	AU	DE	NL	NZ	NO	SE	US	Overall
Preoperative	32.0%	56.0%	48.0%	32.0%	32.0%	44.0%	76.0%	45.7%
Demographics	3/13	6/13	6/13	3/13	6/13	3/13	9/13	
Disease details	5/7	6/7	6/7	5/7	2/7	7/7	6/7	
Surgery preparation details	0/5	2/5	0/7	0/5	0/5	1/5	4/5	
Intraoperative	37.5%	50.0%	37.5%	37.5%	18.8%	31.3%	25.0%	33.9%
Surgery details	3/10	5/10	3/10	3/10	2/10	3/10	1/10	
Intraoperative pitfalls	1/3	1/3	1/3	1/3	0/3	1/3	1/3	
Intraoperative difficulty	2/3	2/3	2/3	2/3	1/3	1/3	2/3	
Early post‐operative	62.5%	31.3%	75.0%	62.5%	43.8%	50.0%	37.5%	51.8%
Discharge	4/6	1/6	5/6	4/6	4/6	5/6	4/6	
Anastomotic complications	6/10	4/10	7/10	6/10	3/10	3/10	2/10	
Long‐term post‐operative	18.2%	4.5%	9.1%	18.2%	0%	59.1%	0%	15.6%
Anastomosis status	0/4	0/4	0/4	0/4	0/4	3/4	0/4	
Complications	2/13	1/13	2/13	2/13	0/13	6/13	0/13	
Functional outcomes	0/3	0/3	0/3	0/3	0/3	2/3	0/3	
Oncological outcomes	2/2	0/2	0/2	2/2	0/2	2/2	0/2	
Total	35.4%	35.4%	40.5%	35.4%	22.8%	46.8%	36.7%	36.2% *p* = 0.003

*Note*: *p* values <0.05 are considered statistically significant.

Abbreviations: AU, Australia; DE, Denmark; NL, Netherlands; NZ, New Zealand; NO, Norway; SE, Sweden; US, United States.

Registries reported a median of 11 out of 25 preoperative variables (range 8–19, IQR 6) (Supplement 1). Among these, gender, American Society of Anaesthesiologists (ASA) classification, and body mass index (BMI) were the only demographic variables universally recorded. For disease‐related characteristics, neoadjuvant therapy and the type of neoadjuvant therapy were consistently documented. No variables related to preoperative surgical bowel preparation were universally captured.

Out of the intraoperative elements, a median of 6 of 16 were reported (range 3–8, IQR 2) (Supplement 2). The variables universally reported were conversion to open surgery and the distance from the anal verge in rectal tumours. No registry included information on the number of staple loads, intraoperative perfusion assessment using fluorescence angiography, anastomotic integrity testing or the outcomes and consequences of such tests. Similarly, variables related to the planning of diverting stoma creation, pelvic staple failure and other device‐related failures were not reported in any registry.

For early post‐operative outcomes, a median of 8 out of 16 variables was captured (range 5–12, IQR 4) (Supplement 3). Mortality, anastomotic complication and reinterventions were the only variables consistently recorded across all registries. In contrast, no registry documented serial C‐reactive protein (CRP) measurements, nor outcomes or consequences of the diagnostic modalities used to detect anastomotic complications.

Moreover, none of the long‐term variables were universally captured across registries, with a median of 2 out of 22 variables recorded (range 0–13, IQR 4) (Supplement 4). Only Australia (4/22), Denmark (1/22), the Netherlands (2/22), New Zealand (4/22) and Sweden (13/22) reported on outcomes beyond the 90‐day post‐operative period.

## DISCUSSION AND CONCLUSIONS

The present study highlights a critical gap in the global infrastructure for monitoring CAL. A key finding is that the majority of countries do not maintain a national registry that systematically captures CAL‐related data. This absence of structured reporting mechanisms limits the ability to accurately quantify the incidence of CAL, evaluate trends over time and benchmark outcomes across institutions or healthcare systems. As a result, opportunities for quality improvement and evidence‐based standardization remain underdeveloped.

Although the collection of data within clinical registries does not directly translate into immediate changes in clinical practice, it is closely associated with improvements in clinical awareness and decision‐making. Several studies have demonstrated that registry‐based quality improvement initiatives are associated with increased compliance with clinical guidelines and changes in clinical practice [19, 20]. The systematic recording of evidence‐based variables enforces their perceived importance and facilitates a more structured approach to patient assessment and management [19]. Additionally, previous research has suggested that repeated exposure to specific variables can enhance cognitive recall through subconscious reinforcement [21, 22]. As clinicians are constantly prompted to document particular variables, they become more likely to recognize and consider these elements during routine clinical practice. This process may contribute to more comprehensive risk assessment, improved adherence to best practices and ultimately better patient outcomes, even in the absence of formal interventions directly linked to the registry itself.

Only 18.4% of the countries included in this study maintained a national registry capturing CAL and CAL‐related variables. In countries without such registries, alternative data collection structures were occasionally present, although these varied widely in scope and completeness. For example, in Argentina, most hospitals participate in a national scientific study that collects relevant CAL‐related variables [23]. However, participation is not mandatory and does not yet include all hospitals nationwide, thereby introducing potential selection bias in the reported outcomes. Similarly, in the United Kingdom, hospitals are required to contribute to a national registry focused on oncological outcomes [24]. While this registry mandates data entry at a national level, it only captures a limited set of post‐operative outcomes and does not include detailed information on post‐operative complications such as CAL.

Even among countries with established national surgical registries, our findings demonstrate considerable heterogeneity in the variables that are collected and reported. This variability was observed across the preoperative, perioperative and post‐operative periods. Such inconsistency limits meaningful comparison between data sets and reduces the overall utility of registries for clinical research, benchmarking and quality assurance.

The assessment of preoperative and intraoperative risk factors revealed incomplete reporting of key variables known to influence the risk of CAL, including patient comorbidities, nutritional status, operative approach and anastomotic technique [25, 26]. This lack of standardization impairs adequate risk stratification at the individual patient level. Furthermore, information regarding preoperative counselling and the potential need for stoma formation during surgery is not systematically captured across registries. Such data are clinically relevant, as previous studies have shown that adequate preoperative preparation is associated with improved post‐operative outcomes, not only in terms of technical success but also with respect to patient‐reported quality of life and satisfaction with their care [27, 28].

In addition, data on intraoperative difficulty and adverse events were scarcely reported, with virtually no registries systematically documenting perioperative anastomotic testing. This is notable given accumulating evidence supporting the potential added value of intraoperative techniques such as indocyanine green (ICG) fluorescence angiography in reducing anastomotic complications in left‐sided anstomoses [29, 30, 31]. The limited incorporation of such variables into registry data sets may reflect the delay between the emergence of scientific evidence in the literature and its translation into routine clinical practice and subsequent implementation in national databases.

Moreover, substantial variation was observed in the reporting of CAL‐related outcomes. While many registries primarily focus on short‐term post‐operative outcomes, the assessment of outcomes beyond 90 days was notably limited. Contemporary registry frameworks that do include long‐term follow‐up tend to prioritize oncological outcomes, with comparatively less emphasis on surgical and functional outcomes. This represents an important, as CAL may result in prolonged clinical consequences that extend well beyond the immediate post‐operative period. Insufficient long‐term follow‐up, therefore, restricts a comprehensive understanding of the true burden of CAL, including its impact on patient recovery, quality of life and long‐term morbidity.

The heterogeneity identified in this study reflects a broader lack of consensus regarding what constitutes essential data for CAL reporting [32, 33]. Without a standardized framework, registries evolve independently, often shaped by local priorities and resource constraints. While this flexibility may be advantageous in certain contexts, it ultimately delays the development of a cohesive global data set that can drive large‐scale analyses and establish best practices. To address these challenges, we propose the global adoption of the CoReAL reporting framework into national registries for colorectal surgery [12]. By promoting uniform data collection, CoReAL has the potential to enhance comparability across registries, facilitate high‐quality research and support more informed clinical decision‐making.

A strength of this study was the benchmarking of variables included in the registries against the CoReAL framework, which is based on consensus from a large international, multidisciplinary panel of experts and incorporates the most up‐to‐date scientific evidence alongside patient perspectives [34]. This provides a robust and clinically relevant reference standard for assessing the completeness and quality of reported variables. Additionally, the study employed a systematic and global approach to identifying registries, engaging surgeons from multiple regions through publications, conferences and professional networks to obtain comprehensive and representative data.

However, several limitations should be acknowledged. The selection of participating surgeons was subject to bias, with underrepresentation of surgeons from low‐ and middle‐income countries. Surgeons from these regions were more difficult to identify through mainstream publications and established surgical networks. Although efforts were made to broaden recruitment strategies through multiple outreach channels, some geographic regions and practice settings remain insufficiently represented. Furthermore, the study relied on self‐reported information from participants, which introduces the potential for reporting inaccuracies. To improve data reliability, registry information was verified whenever possible through registry manuals, publicly available documentation, official websites and consultation with multiple representatives from the same country.

Another limitation of this study is that all investigated registries included in this study primarily collect data on patients undergoing colorectal resections for malignant diseases, rather than for benign indications. This likely reflects the greater availability of funding and infrastructure for oncological research and quality initiatives. Registries included in this study slightly differ in terms of their organizational structure, participation requirements and degree of national coverage. We included all six registries, despite these differences, as they are all established nationwide registries that collect data from hospitals across their respective countries and do not exclude specific geographical regions. Furthermore, participation rates are substantial, allowing these registries to provide a representative overview of the current clinical practice within their healthcare systems. Finally, the dynamic nature of registries should be remarked, since databases are constantly evolving, and the variables assessed at the time of data collection may not fully reflect future registry content.

Future research should focus on the global implementation and validation of standardized reporting frameworks such as the CoReAL framework to improve consistency and comparability of CAL‐related data. Moreover, future research should evaluate the additional time and resources required for implementation of the CoReAL reporting framework. Efforts are also needed to support the development and integration of registries in underrepresented regions, ensuring more equitable global data coverage. Integrating such frameworks directly into electronic patient files is essential, as this may reduce administrative burden while simultaneously improving the completeness and accuracy of data collection. Because data are typically collected at the institutional level, complications diagnosed and treated at another hospital after discharge may not be captured unless patient records are linked across institutions, which emphasizes the need for uniform reporting. Ultimately, linking high‐quality registry data to clinical outcomes may facilitate targeted interventions and drive continuous improvements in colorectal surgical care.

In conclusion, the current lack of standardization in the reporting of CAL‐related elements undermines robust risk stratification methods and limits the clinicians' ability to tailor surgical strategies to individual patients. Overall, CAL reporting is currently characterized by limited global coverage and substantial variability across existing registries. The implementation of a standardized reporting framework represents a crucial step towards improving data quality, allowing meaningful comparisons and ultimately enhancing patient outcomes in colorectal surgery.

## AUTHOR CONTRIBUTIONS

**Anne de Wit:** Conceptualization; data curation; validation; formal analysis; investigation; writing – original draft; writing – review and editing; project administration; methodology; visualization. **Freek Daams:** Data curation; supervision; project administration; writing – review and editing. **Katherine Lam:** Conceptualization; data curation; writing – review and editing. **Katherine Donovan:** Conceptualization; data curation; writing – review and editing. **Daniela Ramos‐Delgado:** Data curation; writing – review and editing. **Danique J. I. Heuvelings:** Data curation; writing – review and editing. **Nicole D. Bouvy:** Data curation; supervision; writing – review and editing. **Nader Francis:** Data curation; supervision; writing – review and editing. **Marylise Boutros:** Data curation; supervision; writing – review and editing. **Patricia Sylla:** Conceptualization; data curation; validation; formal analysis; investigation; writing – original draft; supervision; project administration; methodology.

## FUNDING INFORMATION

There was no financial support provided by a third party.

## CONFLICT OF INTEREST STATEMENT

FD received an educational grant from Medtronic. NB is a member of the advisory board of Active Surgical and received an educational grant from Medtronic. PS received consulting fees from Medtronic, Ethicon, Safeheal, Vertex and Olympus. All other authors declare no conflict of interests.

## ETHICS STATEMENT

Ethical approval was granted by the Ethical Committee of the Mount Sinai Hospital and exempted from the Medical Research Involving Human Subjects Act (WMO). This study adhered to the Declaration of Helsinki.

## ARTIFICIAL INTELLIGENCE STATEMENT

ChatGPT was used solely to improve the language and readability of the manuscript and to assist in identifying grammatical and spelling errors. It was not used to generate study data, perform analysis, interpret results or create tables or figures. All scientific content, including the study design, data interpretation and conclusions, were developed by the authors. Following language editing by AI, all authors critically reviewed, revised and approved the final version of the manuscript.

## Supporting information


**Supplement 1.** Preoperative elements.
**Supplement 2**. Intraoperative elements.
**Supplement 3**. Post‐operative elements.
**Supplement 4**. Long‐term post‐operative elements.

## Data Availability

The data that support the findings of this study are available from the corresponding author upon reasonable request.
