# Supplementary material for: Reporting of colorectal anastomotic leakage: Worldwide audit of national databases
Source: Colorectal Dis. 2026 Sep 7;28(9):e70619. doi: 10.1111/codi.70619 (PMC13550949; doi:10.1111/codi.70619)
Supplement: Supplementary file 1 [file CODI-28-0-s001.docx]

**SUPPLEMENT 1** – Preoperative elements

| **Elements** | **Countries that collected the variable** | |
| --- | --- | --- |
| *Demographics* |  |  |
| Gender | 7/7 | AU, DE, NL, NZ, NO, SE, US |
| ASA score | 7/7 | AU, DE, NL, NZ, NO, SE, US |
| BMI | 7/7 | AU, DE, NL, NZ, NO, SE, US |
| Alcohol | 1/7 | DE |
| Drinks per week | 1/7 | DE |
| Active smoker | 2/7 | DE, US |
| Cigarettes per day | 0/7 | - |
| Serum albumin level | 2/7 | NO, US |
| Diabetes mellitus | 3/7 | NL, NO, US |
| HbA1c level | 1/7 | US |
| Cardiovascular disease | 3/7 | NL, NO, US |
| Renal failure | 2/7 | NL, US |
| Chronic steroid use | 1/7 | US |
| *Disease details* |  |  |
| T-stage | 6/7 | AU, DE, NL, NZ, SE, US |
| Tumor size | 1/7 | SE |
| Tumor location | 6/7 | AU, DE, NL, NZ, SE, US |
| Tumor perforation | 4/7 | DE, NL, SE, US |
| Tumor obstruction | 6/7 | AU, DE, NL, NZ, SE, US |
| Neoadjuvant therapy | 7/7 | AU, DE, NL, NZ, NO, SE, US |
| Type of neoadjuvant therapy | 7/7 | AU, DE, NL, NZ, NO, SE, US |
| *Surgery preparation details* |  |  |
| Potential need for stoma discussed | 1/7 | US |
| Referral to stoma therapist | 1/7 | US |
| Antibiotic prophylaxis | 2/7 | DE, US |
| Type of antibiotics | 0/7 | - |
| Mechanical bowel preparation | 3/7 | DE, SE, US |
| AU = Australia; DE = Denmark; NL = Netherlands; NZ = New Zealand; NO = Norway; SE = Sweden; US = United States.  ASA = American Society of Anesthesiologists; BMI = Body Mass Index. | | |

**SUPPLEMENT 2** – Intraoperative elements

| **Elements** | **Countries that collected the variable** | |
| --- | --- | --- |
| *Surgery details* |  |  |
| Conversion to open surgery | 7/7 | AU, DE, NL, NZ, NO, SE, US |
| Location of IMA ligation | 2/7 | DE, SE |
| Splenic flexure mobilization | 1/7 | DE |
| Number of staple loads | 0/7 | - |
| Perfusion assessment with fluorescence angiography | 0/7 | - |
| Anastomotic integrity testing | 0/7 | - |
| Outcome and consequence | 0/7 | - |
| Diverting stoma creation | 6/7 | AU, DE, NL, NZ, NO, SE, US |
| Planned | 0/7 | - |
| Stoma type | 4/7 | AU, DE, NL, NZ |
| *Intraoperative pitfalls* |  |  |
| Unplanned multivisceral resection | 6/7 | AU, DE, NL, NZ, SE, US |
| Pelvic staple failure | 0/7 | **-** |
| Other device failure | 0/7 | **-** |
| *Intraoperative difficulty* |  |  |
| Distance from anal verge | 7/7 | AU, DE, NL, NZ, NO, SE, US |
| Acute blood loss requiring blood transfusion | 2/7 | DE, US |
| Redo pelvic surgery | 3/7 | AU, NL, NZ |
| AU = Australia; DE = Denmark; NL = Netherlands; NZ = New Zealand; NO = Norway; SE = Sweden; US = United States.  IMA = Inferior mesenteric artery. | | |

**SUPPLEMENT 3** – Postoperative elements

| **Elements** | **Countries that collected the variable** | |
| --- | --- | --- |
| *Discharge* |  |  |
| Serial CRP measurements | 0/7 | - |
| Length of hospital stay | 6/7 | AU, NL, NZ, NO, SE, US |
| Length of intensive care unit stay | 2/7 | NL, SE |
| Readmission | 6/7 | AU, NL, NZ, NO, SE, US |
| Mortality | 7/7 | AU, DE, NL, NZ, NO, SE, US |
| Date of death | 6/7 | AU, NL, NZ, NO, SE, US |
| *Anastomotic complications* |  |  |
| Anastomotic complication | 7/7 | AU, DE, NL, NZ, NO, SE, US |
| Diagnostic modality | 2/7 | AU, NZ |
| Date | 1/7 | DE |
| Outcome and consequence | 0/7 | - |
| Reinterventions | 7/7 | AU, DE, NL, NZ, NO, SE, US |
| Date | 4/7 | AU, NL, NZ, SE |
| Outcome and consequence | 4/7 | AU, NL, NZ, NO |
| Stoma creation | 4/7 | AU, DE, NL, NZ |
| Stoma type | 1/7 | NL |
| Stoma diversion | 1/7 | NL |
| AU = Australia; DE = Denmark; NL = Netherlands; NZ = New Zealand; NO = Norway; SE = Sweden; US = United States.  CRP = C-reactive protein. | | |

**SUPPLEMENT 4** – Long-term postoperative elements

| **Elements** | **Countries that collected the variable** | |
| --- | --- | --- |
| *Anastomosis status* |  |  |
| Healed anastomosis | 0/7 | - |
| Stoma closure | 1/7 | SE |
| Stoma in place | 1/7 | SE |
| Reason for stoma in place | 1/7 | SE |
| *Complications* |  |  |
| Readmission | 0/7 | - |
| Anastomotic complication | 1/7 | SE |
| Diagnostic modality | 0/7 | - |
| Date | 0/7 | - |
| Outcome and consequence | 0/7 | - |
| Reinterventions | 1/7 | SE |
| Date | 0/7 | - |
| Outcome and consequence | 0/7 | - |
| Stoma creation | 1/7 | SE |
| Stoma type | 1/7 | SE |
| Stoma diversion | 0/7 | - |
| Mortality | 5/7 | AU, DE, NL, NZ, SE |
| Date of death | 4/7 | AU, NL, NZ, SE |
| *Functional outcomes* |  |  |
| LARS score | 1/7 | SE |
| EQ-5D score | 1/7 | SE |
| Wexner FI score | 0/7 | - |
| *Oncological outcomes* |  |  |
| Local recurrence | 3/7 | AU, NZ, SE |
| Distant metastasis | 3/7 | AU, NZ, SE |
| AU = Australia; DE = Denmark; NL = Netherlands; NZ = New Zealand; NO = Norway; SE = Sweden; US = United States. | | |
